# Clara Cell 10 kDa Protein Alleviates Murine Hepatitis Virus Strain 3-Induced Fulminant Hepatitis by Inhibiting Fibrinogen-Like Protein 2 Expression

**DOI:** 10.3389/fimmu.2018.02935

**Published:** 2018-12-13

**Authors:** Haijing Yu, Yang Liu, Hongwu Wang, Xiaoyang Wan, Jiaquan Huang, Weiming Yan, Dong Xi, Xiaoping Luo, Guanxin Shen, Qin Ning

**Affiliations:** ^1^Department of Infectious Diseases, Tongji Hospital, Tongji Medical College, Huazhong University of Science and Technology, Wuhan, China; ^2^Department of Otolaryngology-Head and Neck Surgery, Tongji Hospital, Tongji Medical College, Huazhong University of Science and Technology, Wuhan, China; ^3^Department of Pediatrics, Tongji Hospital, Tongji Medical College, Huazhong University of Science and Technology, Wuhan, China; ^4^Department of Immunology, School of Basic Medicine, Tongji Medical College, Huazhong University of Science and Technology, Wuhan, China

**Keywords:** CC10, Fgl2, hepatitis, MHV-3, macrophage

## Abstract

**Background:** Fulminant hepatitis (FH) is a serious threat to human life, accompanied by massive and rapid necroinflammation. Kupffer cells, the major immune cell population involved in innate immune responses, are considered to be central for FH. Fibrinogen-like protein 2 (Fgl2) is a pro-coagulant protein that is substantially induced in macrophages upon viral infection, and Fgl2 depletion represses murine hepatitis virus strain 3 (MHV-3) infection. Clara cell 10 kDa (CC10) protein is a secretory protein with anti-inflammatory properties in allergic rhinitis and asthma. However, its mechanisms of action and pathogenic roles in other disease are still unclear. In this study, we aimed to determine the role of CC10 in FH and the regulation of Fgl2 by CC10.

**Methods:** A mouse FH model was established by peritoneal injection of MHV-3. The mice received CC10 protein through tail vein injection before viral infection. Survival rate, liver function, liver histology, fibrin deposition, and necrosis were examined. The regulatory effect of CC10 on Fgl2 expression was investigated using THP-1 cells and mouse peritoneal macrophages *in vitro*.

**Results:** In the mouse FH model induced by MHV-3, the survival rate increased from 0 to 12.5% in the CC10 group compared to that in the saline-only control group. Meanwhile, the levels of ALT and AST in serum were significantly decreased and liver damage was reduced. Furthermore, hepatic Fgl2, TNF-α, and IL-1β expression was obviously downregulated together with fibrin deposition, and hepatocyte apoptosis was reduced after administration of CC10 protein. *In vitro*, CC10 was found to significantly inhibit the expression of Fgl2 in IFN-γ-treated THP-1 cells and MHV-3-infected mouse peritoneal macrophages by western blot and real-time PCR. However, there was no direct interaction between CC10 and Fgl2 as shown by co-immunoprecipitation. Microarray investigations suggested that HMG-box transcription factor 1 (HBP1) was significantly low in CC10-treated and IFN-γ-primed THP-1 cells. HBP1-siRNA treatment abrogated the inhibitory effect of CC10 on Fgl2 expression in Human Umbilical Vein Endothelial cells (HUVECs).

**Conclusion:**CC10 protects against MHV-3-induced FH via suppression of Fgl2 expression in macrophages. Such effects may be mediated by the transcription factor HBP1.

## Introduction

Fulminant hepatitis (FH) is a serious life-threatening disease characterized by massive hepatocyte necrosis, severe liver damage, and high mortality. The underlying mechanisms and the pathogenesis of FH are not clear. However, accumulating evidence suggests that, regardless of the pathogenesis of FH, the host's inflammatory responses contribute to liver microcirculatory disorders and injuries. Accordingly, It has been shown that immune cell activation and inflammatory cytokines play an important role in FH (1). In recent years, our laboratory has conducted extensive research on the pathogenesis of FH and found that immune cells play a key role in it. Kupffer cells, natural killer (NK) cells (2, 3), cytotoxic T-lymphocytes (CTLs), and double negative T-cells (DNT) (4–6) in liver and the cytokines that are produced by these cells cause liver damage.

Prothrombinase Fgl2 belongs to the fibrinogen superfamily and is produced by activated macrophages or endothelial cells, transforming prothrombin directly into thrombin, so as to quickly initiate the process of coagulation. This promotes the conversion of fibrinogen into fibrin, resulting in thrombosis (7–12). Our study found that Fgl2 was highly expressed in peripheral blood mononuclear cells (PBMCs) and in liver tissue of humans or mice with severe viral hepatitis, and was positively related to the severity of the disease (13, 14). Gene therapy targeting Fgl2 silencing showed that the survival rate of fulminant hepatitis mice increased from 0 to 33.3% (15). Thus far, the discovery and related research involving Fgl2 have provided new insights into the molecular mechanism of hepatocyte necrosis in FH. In view of the important role of Fgl2 in severe viral hepatitis, investigations concerning the regulation of Fgl2 will be beneficial in the search for new strategies for treatment of severe hepatitis.

Clara cell 10 kDa protein (CC10), also considered to be uteroglobin, Clara cell secretory protein, is one of members of secretoglobin superfamily. Expressed in mucosal epithelial cells of organs (including lungs and nose) that communicated with the outside world (16). CC10 has immunomodulatory and anti-inflammatory effects. Compared to wild-type mice, CC10-knockout mice exhibited excessive airway inflammation caused by allergic reaction and bacterial and viral infections (17). Reduced levels of CC10 are associated with inflammatory and allergic airway diseases, including sinusitis, asthma and allergic rhinitis (18–21).

Previous studies and published articles show that CC10 protein can not only inhibit Th17 cell responses by inhibiting expression of related molecules of dendritic cells and cytokines in mice with allergic rhinitis, but also can inhibit chitosan-3 like protein 1 (22, 23). Moreover, CC10 inhibits the expression of an important immune regulator, osteopontin (OPN), in models of allergic rhinitis (21).

In this study, we investigated the role of CC10 in hepatitis virus strain 3 (MHV-3)-induced FH in mice and explored whether CC10 protein could regulate Fgl2 in the disease process.

## Materials and Methods

### Mice

Female BALB/cJ mice (Shanghai Shilaike Animal Seed Center, Shanghai, China), 6–8 weeks of age, with a body weight of 18.0–20.0 g, were kept in Tongji Hospital with food and water. Mice were divided into two groups: CC10 group (experimental group) and phosphate-buffered saline (PBS) group (control group). This study was carried out in accordance with the recommendations of the guidelines of the National Institutes of Health and the Animal Experiment Committee of Tongji hospital. This study was reviewed and approved by the Animal Experiment Committee of Tongji hospital.

### Cells and Culture Conditions

The human monocyte cell line THP-1 was purchased from the Cell Institute of the Chinese Academy of Sciences (Shanghai, China). Human Umbilical Vein Endothelial Cells (HUVECs) were obtained from the Biology Treasure Center of Wuhan University, China. The Chinese hamster ovary (CHO) cell line was acquired from the typical culture preservation commission cell bank, the Chinese Academy of Sciences (Shanghai, China). Human Umbilical Vein Endothelial Cells (HUVECs) and CHO cells were cultured in Dulbecco's modified Eagle's medium (DMEM), and THP-1 cells were maintained in RPMI 1,640 containing 10% heat inactivated fetal bovine serum (FBS, Gibco Life Technologies, USA), 100 U/mL penicillin, and 100 mg/mL streptomycin and cultured at 37°C, 50 mL/L CO_2_ and 95% humidity.

### PEMs Isolation and Culture

Peritoneal exudative macrophages (PEMs) were obtained from BALB/cJ mice. Cells were resuspended in RPMI 1,640 supplemented with 10% FBS at 1–2 × 10^6^ cells/mL in a 6-well plate and incubated for 4 h. They were then washed with RPMI 1640 medium and non-adherent cells discarded. The adherent cells were macrophages and were incubated for a further 12 h. Peritoneal exudative macrophages (PEMs) were divided into two groups. One group was supplemented with CC10 protein (150 ng/mL) and in the other group, PBS was added. After 2 h of stimulation, 1,000 plaque forming units (PFUs) of MHV-3 was added to the cells, which were then cultured for 4 h. Peritoneal exudative macrophages (PEMs) were harvested and lysed for real-time PCR and western blotting analysis.

### Detection of Hepatocyte Apoptosis Using TUNEL Assay

Cell apoptosis was detected by the terminal deoxynucleotidyl transferase dUTP nick end labeling (TUNEL) method with a TUNEL apoptosis detection kit (Roche, Switzerland). Briefly, 5 μm sections were deparaffinized, dehydrated through an alcohol series and incubated with proteinase K for 30 min at 37°C. After stopping the proteinase K digestion reaction with PBS, the samples were incubated with terminal deoxynucleotidyl transferase end-labeling cocktail (a mixture of terminal deoxynucleotidyl transferase and dUTP at a ratio of 2:29, respectively), for 2 h at 37°C in an immunohistochemistry wet box. Following washing and blocking, each section was supplemented with reagent (converter-POD) to cover the tissues and incubated for 30 min at 37°C in a wet box. Then, the liver tissue sections were washed with PBS, and colored with diaminobenzidine (DAB) subsequently. Hepatocytes with nucleus stained brownish yellow were considered to be apoptotic cells.

### Flow Cytometry

The expression of Fgl2 on THP-1 cells was measured by flow cytometry (BD FACS Canto II, USA). Briefly, cells (2 × 10^5^ per tube) were incubated with Human TruStrain FcX (Fc Receptor Blocking solution, BioLegend, USA) for 10 min at room temperature and then incubated in the dark with mouse anti-Fgl2 antibody (1:100, Abnova,) or normal goat serum (an isotype control) at 4°C for 40 min. Cells were washed with PBS and incubated in the dark with PE-conjugated goat anti-mouse IgG antibody (1:50, BioLegend, USA) at 4°C for 30 min. Cells were then washed with PBS and resuspended in 300 μL PBS for study.

### Histology and Immunohistochemistry

Liver slices were fixed in 4% paraformaldehyde and then embedded in paraffin. Immunohistochemistry of liver tissues was performed using SP-9001 SPlink Detection Kits (Biotin-Streptavidin HRP Detection Systems) (ZSGB-BIO, Beijing, China) according to the manufacturer's instructions. For immunohistochemistry staining, the expression of Fgl2, fibrinogen, Fas and TNF-receptor 1 in mouse liver tissues was detected with polyclonal rabbit anti-mouse Fgl2 antibody (1:100, Proteintech, USA), polyclonal rabbit anti-mouse fibrinogen antibody (1:1,000, Abcam, EngLand), polyclonal rabbit anti-mouse Fas antibody (1:50, Abcam, EngLand), and polyclonal rabbit anti-mouse TNF-receptor 1 antibody (1:500, Abcam, EngLand), respectively. After incubation with an horseradish peroxidase (HRP)-labeled goat IgG fraction to rabbit IgG Fc, the target protein was detected using a DAB kit (ZSGB-BIO, Beijing, China). The slides were then counterstained with hematoxylin and visualized under a microscope (Olympus, Tokyo, Japan).

### Western Blotting

Liver tissue and cells were homogenized in RIPA lysis buffer with phenyl methane sulfonyl fluoride (PMSF) protease inhibitor. Protein lysates were separated by SDS-PAGE, and western blotting was performed using a monoclonal mouse anti-human/mouse Fgl2 (1:750, Abnova), a monoclonal mouse anti-human HBP1 (1:100, Santa Cruz, USA), and a monoclonal rabbit anti- human/mouse β-actin (1:1,000, Cell Signaling Technology, USA).

### Real-Time Quantitative PCR (qPCR)

Liver tissues were collected from MHV-3-infected BALB/cJ mice at 72 h, and total RNA was extracted using Trizol Reagent (Invitrogen, USA) and then reverse transcribed into cDNA by using ReverTra Ace qPCR RT kit (TOYOBO, Japan). The cDNA was then amplified by RT-PCR by using Dream Taq Green PCR Master Mix (2 ×) (Thermo Scientific, USA). Real-time quantitative PCR (qPCR) with SYBR Green Real-time PCR Master Mix (TOYOBO, Japan) was performed using a CFX96 real-time PCR detection system (Bio-Rad, USA) and mRNA levels were normalized with reference to those of the house keeping gene *GAPDH*. Primer sequences for qPCR amplification were as follows: mTNF-α forward, 5′-TTT GAG ATC CAT GCC GTT GG-3′; mTNF-α reverse, 5′-GCCA CCA CGC TCT TCT GT-3′; mIL-1β forward, 5′-TGT AAT GAA AGA CGG CAC ACC- 3′; mIL-1β reverse, 5′-TCT TCT TTG GGT ATT GCT TGG-3′. mFgl2 forward, 5′-GCC AAA TGT GAG TCC CTG GAA-3′; mFgl2 reverse, 5′-TTC CAC CCA AGA GCA CGT TTA AG-3′; hFgl2 forward 5′-ACA GTT CAG GCT GGT GGT-3′; hFgl2 reverse, 5′-GGC TTA AAG TGC TTG GGT-3′; HBP1 forward, 5′-TGA AGC AGA AGC TGG GAGT-3′; HBP1 reverse, 5′-GGC TCT TAG GCT GGG ACA-3′; GAPDH forward, 5′-CGG ATT TGG TCG TAT TGGG-3′; GAPDH reverse, 5′-CTC GCT CCTG GAAG ATGG-3′.

### Cytokine Treatment

THP-1 cells were treated with 100 ng/ml phorbol 12-myristate 13-acetate (PMA) (Sigma, USA) for 48 h to induce differentiation toward adherent macrophage-like cells as reported previously (24). The CC10 group was supplemented with CC10 protein (150 ng/ml). After 2 h of stimulation, IFN-γ (10 ng/ml) was added to these cells, which were then cultured for 12 h before they were collected for western blotting and real-time PCR studies.

### Transfection

The Chinese hamster ovary (CHO) cells were cultured in 10 cm cell culture dishes with DMEM supplemented with 10% FBS until 80–90% confluence. Next, 12 μg pcDNA3.1-hFgl2 (constructed in our lab) was mixed with 12 μg pcDNA3.1-hCC10 in serum-free DMEM. The mixture was then combined with Lipofectamine 2,000 (Invitrogen, USA) and mixed gently. After incubation at 27°C for 20 min, the solution was added to CHO cells and incubated at 37°C in 5% CO_2_. Four to Six hour after transfection, the medium was removed and fresh medium containing 10% FBS was added. At 48 h after transfection, the cells were collected for co-immunoprecipitation analysis to evaluate the interaction of CC10 with Fgl2.

Both HUVEC and THP-1 cells express fgl2. However, in the transfection experiments, it is difficult to transfect the THP-1 cells with siRNA, so we use HUVEC instead of THP-1. Human Umbilical Vein Endothelial Cells (HUVECs) were cultured in six-well plates with DMEM supplemented with 10% FBS until 70–80% confluence. 50 pmol HBP1-siRNA was mixed with 125 μl serum-free DMEM. Two microliter Lipofectamine 2,000 was gently mixed with serum-free DMEM. After incubation at 27°C for 5 min, the solution was added to HUVECs and incubated at 37°C. Four hour after transfection, the medium was removed and fresh medium containing 10% FBS was added. At 48 h after transfection, cells were collected for real-time PCR and western blot analysis to evaluate the effects of HBP1 on Fgl2. At 24 h after transfection, the CC10 group was supplemented with the CC10 protein (150 ng/mL). After 4 h of stimulation, IFN-γ (10 ng/mL) was added to these cells. These cells were then cultured for 24 h before they were harvested for real-time PCR studies to evaluate the effects of CC10 on Fgl2 by HBP1. Negative control was used as a control.

### Co-immunoprecipitation

To detect whether there was a potential interaction between CC10 protein and Fgl2, CHO cells were transfected with pcDNA3.1-hCC10 and pcDNA3.1-hFgl2 for 48 h. Cells transfected with empty plasmid pcDNA3.1 (mock) were used as negative controls for *CC10* gene transfection. Immunoprecipitation and immunoblotting were performed by using Pierce Co-Immunoprecipitation Kit (Pierce, USA). Total cell proteins were extracted as previously described (25). The proteins were immunoprecipitated by mouse anti-human Fgl2 antibody (1:500, Abnova). For co-immunoprecipitation experiments, western blotting was performed using both rat anti-human uteroglobin/SCGB1A1 Antibody (1:750, R&D, USA) and mouse anti-human Fgl2 antibody (1:500, Abnova). Control isotype rat IgG1 was used as a negative control for primary antibodies.

### CC10 Plasmid Construction

The human *CC10* coding region gene, including a 389 bp sequence, was amplified from homogenized human turbinate tissue by RT-PCR. In this study, the sequences of PCR primers for *CC10* were as follows: hCC10-forward, 5′-CCC TCC ACC ATG AAA CTCG-3′; hCC10- reverse, 5′-TGA GAT GCT TGT GGT TTA TTG AAG-3′. The PCR products were cloned into pEASY-T1 cloning vector (TransGEN, Beijing, China) and then subcloned into HindIII/XbaI site of pcDNA3.1 vector (Invitrogen, USA) to form eukaryotic expression plasmids pcDNA3.1- hCC10.

### Microarray

Microarray analysis was used to screen changes in genome-wide gene expression patterns in THP-1 cells with or without CC10 protein. The changes in over 47,000 human gene expression patterns were assessed using Affymetrix gene microarrays (Human Genome U133 Plus 2.0) (CapitalBio Co.,Ltd., Beijing, China). Three replicates were used for microarrays analysis.

### Statistical Analyses

Data obtained from the experiments are expressed as means ± SEM. Survival curve comparisons were performed with the Log Rank test. Multiple group analyses for data were evaluated by one-way analyses of variance. Analyses of two group results were performed using Student's *t*-test to evaluate the statistical significance of differences. Values of *P* < 0.05 indicated significance.

## Results

### CC10 Improved Survival and Reduced Liver Injury in the Mice of MHV-3-Induced FH

**Figure 1:**
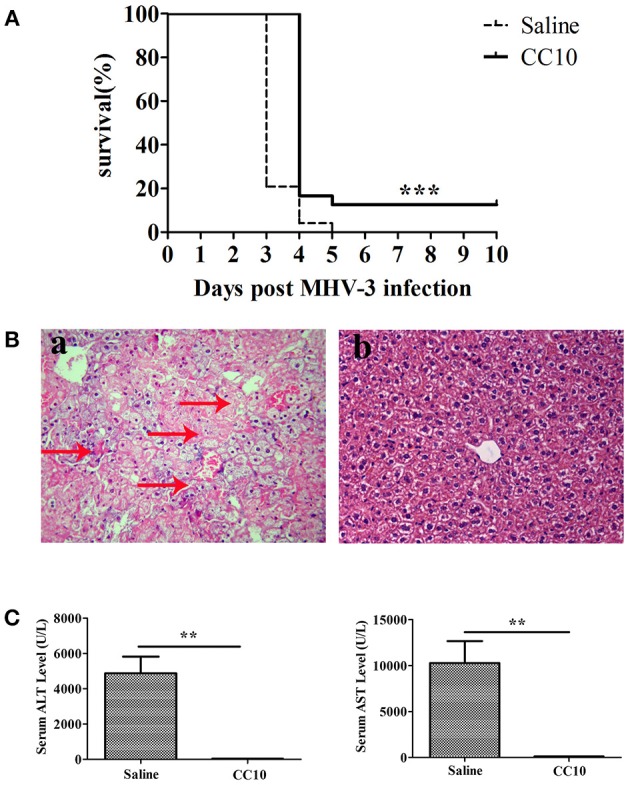
CC10 protein increased survival rate and reduced liver damage in mice. **(A)** The survival rate of CC10 group is higher than the control group comprised of MHV-3–infected BALB/cJ mice treated with saline. CC10 protein (2 μg) or saline were injected into mice by tail vein. BALB/cJ mice then received 100 PFU of MHV-3 intraperitoneally 24 h later to develop fulminant viral hepatitis. Then, CC10 protein (2 μg) or saline were injected into mice by tail vein following MHV-3 infection 24 h later. The survival rate was observed for 10 days (*n* = 24/group). Representative data from three independent experiments are shown. The survival curve was analyzed by using the Log-Rank Test. ***P < 0.001 compared with saline group. **(B)** Histopathology of liver tissues (H&E staining; original magnification, × 400, *n* = 5/group) at 72 h post-MHV-3 infection was evaluated in the two groups of MHV-3–infected BALB/cJ mice. Livers were collected from saline-treated **(a)** and CC10-treated **(b)** BALB/cJ mice at 72 h after MHV-3 infection. Arrows point to inflammatory cell infiltration areas or necrotic regions with inflammation. **(C)** Effect of CC10 on serum ALT and AST levels (*n* = 6–8/group). Values represent means and standard error of three independent experiments performed in triplicate. **P < 0.01 compared with the saline group.

To establish an animal model of mouse FH, MHV-3 was injected intraperitoneally to BALB/cJ mice (24 mice/group). To further study the role of CC10 in FH, recombinant mouse CC10 protein (2 μg/mouse) or saline was administrated into the tail vein 24 h prior to MHV-3 infection. The same dose of CC10 protein or saline was then administered 24 h later. The survival rate of the CC10 and saline groups was observed for 10 days. The results showed that mice in the two groups began to die at 48 h after injection of MHV-3 and exhibited symptoms of horripilation, slow activity, and reduced food consumption. In the CC10 group 24 mice were alive on day 3 after infection, 4 mice alive on day 4, and 3 of 24 (12.5%) mice recovered from fulminant viral hepatitis. At the same time, in saline treated group, there were 5 mice alive on day 3, 1 mice alive on day 4 after infection, and no mice survived to day 5. That is to say, the mice in the saline group died within 3 or 4 days. Three of 24 (12.5%) mice of the CC10 group recovered from fulminant viral hepatitis (Figure 1A).

To better understand the mechanisms underlying the biological effects of the CC10 protein, liver function (ALT and AST levels in serum) and liver histology in mice of MHV-3-infected was performed. Liver tissues were harvested 72 h following MHV-3 infection, and liver histology was detected by H&E staining. These results showed that there was substantial inflammatory cell infiltration and widespread necrosis of hepatocytes in the liver tissue of the saline group mice (Figure 1Ba). There were rare or no infiltrating inflammatory cells, and few or no hepatocyte necrosis in the livers of mice in the CC10 group 72 h after MHV-3 infection (Figure 1Bb). Serum ALT and AST levels in mice were observed 72 h after MHV-3 infection. The results showed that serum ALT and AST levels in the saline group reached a peak 72 h after MHV-3 infection, but there was no significant increase in the CC10 group compared to the levels in the control group (*P* < 0.01, Figure 1C). These results suggested that CC10 protein has a role in protection against MHV-3-induced liver injury in mice.

### CC10-Treated Mice Show Reduced Hepatocyte Apoptosis and Decreased TNF-α and IL-1β Production After MHV-3 Infection

**Figure 2:**
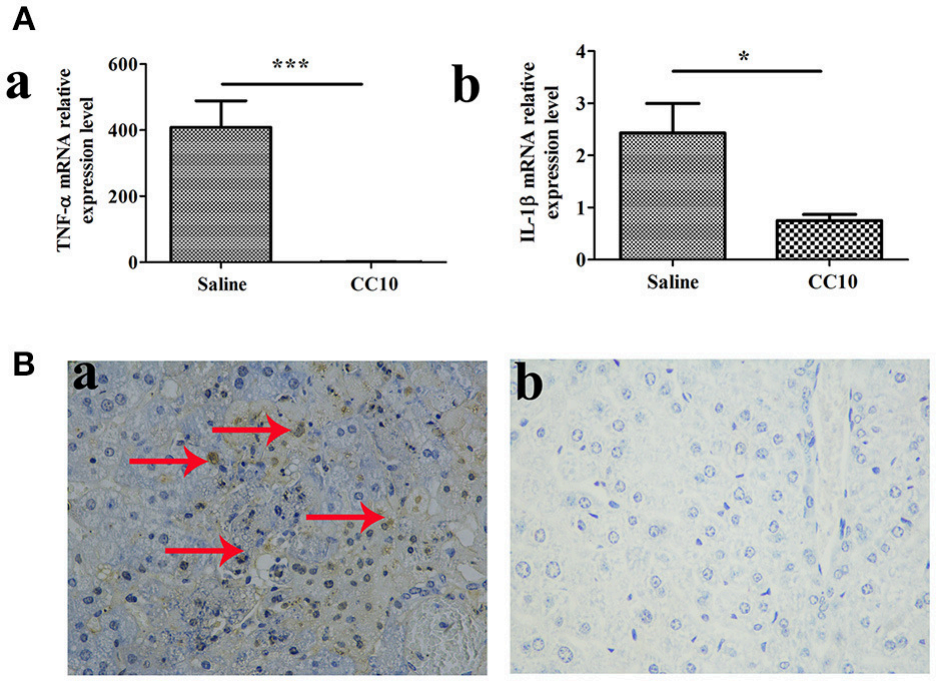
CC10 treatment decreased the expression of TNF-α and IL-1β and inhibited hepatocyte apoptosis in mouse livers. Livers from BALB/cJ mice treated with CC10 protein, and from mice treated with saline were collected 72 h following MHV-3 infection. **(A)** The levels of *TNF-*α **(a)** and *IL-1*β **(b)** in livers were detected using qPCR (*n* = 6/group). Values shown are means and standard error. *P < 0.05 and ***P < 0.001 for CC10 injected vs. saline injected mice. **(B)** Hepatocyte apoptosis was determined by TUNEL. Arrows represent positive staining as shown in brown (Original magnification, × 400). **(a)** Livers were obtained from Saline treated mice; **(b)** Livers were obtained from CC10 treated mice. (*n* = 5/group). Each experiment was repeated in triplicate.

To further elucidate the mechanisms of reduced liver injury following CC10 protein injection, we investigated the cytokines TNF-α and IL-1β expression. Because these two cytokines play a crucial role in the liver damage of FH. They are characterized by an increase in apoptosis. Levels of TNF-α and IL-1β in liver tissues were markedly reduced in the CC10 group (as shown in Figure 2A). Hepatic apoptosis (Figure 2B) was significantly reduced in the CC10 group.

### Expression of Fgl2 and Fibrinogen Was Reduced in CC10-Treated Mice After MHV-3 Infection

**Figure 3:**
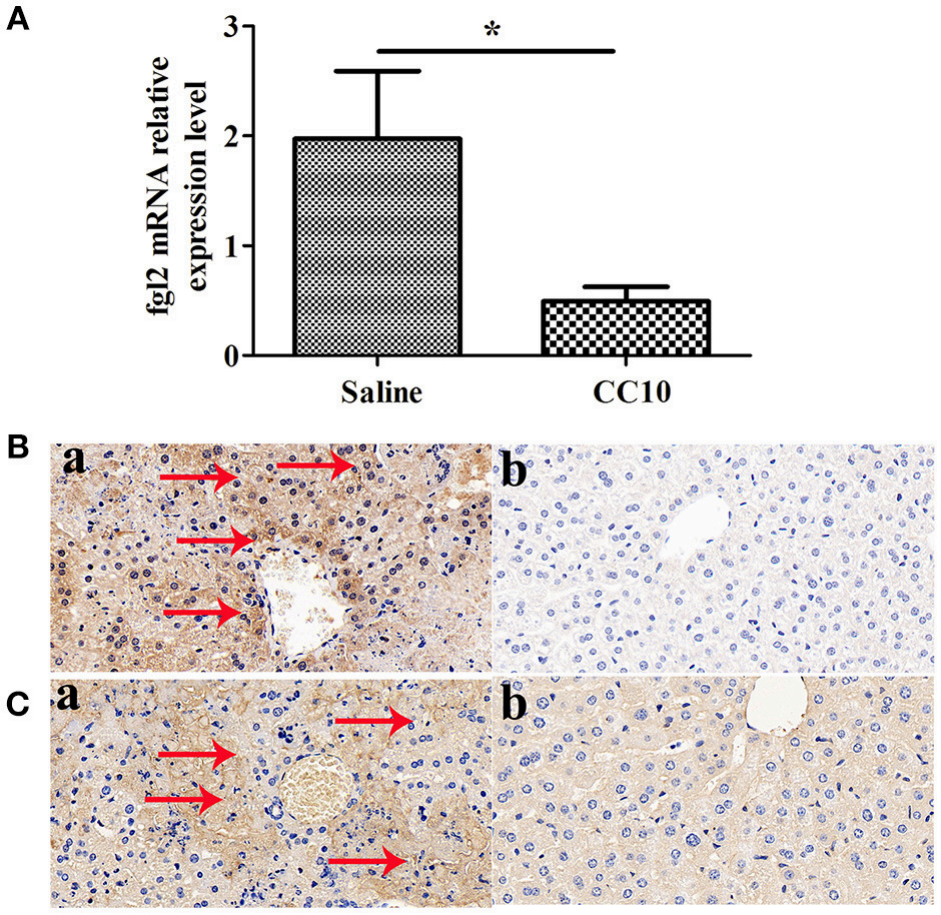
CC10 inhibited Fgl2 expression and decreased fibrin deposition in mice. Livers from BALB/cJ mice treated with CC10 protein, and from mice treated with saline were collected 72 h following MHV-3 infection. **(A)** *Fgl2* expression was measured by qPCR (*n* = 6–8/group). Data represent means and standard error of three independent experiments performed in triplicate. *P < 0.05 compared with the saline group. **(B)** Fgl2 expression and **(C)** fibrin deposition was determined by immunohistochemical analysis. **(a)** Livers were obtained from Saline treated mice; **(b)** Livers were obtained from CC10 treated mice. (*n* = 5/group). Arrows represent positive staining as shown in brown (original magnification × 400). Each experiment was repeated in triplicate.

We and collaborators have a long standing interest in studying the role of fgl2 in viral hepatitis. Fgl2 has been verified to play an essential role in the progression of fulminant viral hepatitis as we appreciate from previous reports. We have provided liver pathology figures and liver function for MHV-3 infected mice with a fgl2 gene knockout as shown in Supplementary Figure 1. The data was comparable with previous reports from our center and collaborators. From this current study we shown that CC10 plays a protective role in liver damage.To study the related molecules of CC10 in MHV-3-induced FH mice, we evaluated whether there was crosstalk between Fgl2 and CC10. We found that the expression of Fgl2 in the liver of mice was reduced 72 h after MHV-3 infection and treatment with CC10 protein (Figures 3A,B). Furthermore, fibrin deposition, an indicator of liver injury associated with Fgl2 expression in FH, was also decreased in the livers of CC10-treated mice compared to that in controls (Figure 3C). This indicates that CC10 treatment reduced liver injury after viral infection by inhibiting Fgl2 expression.

### Recombinant CC10 Protein Inhibits IFN-γ-Induced Fgl2 Expression in THP-1 Cells and Reduces MHV-3-Induced Fgl2 Expression in PEMs

**Figure 4:**
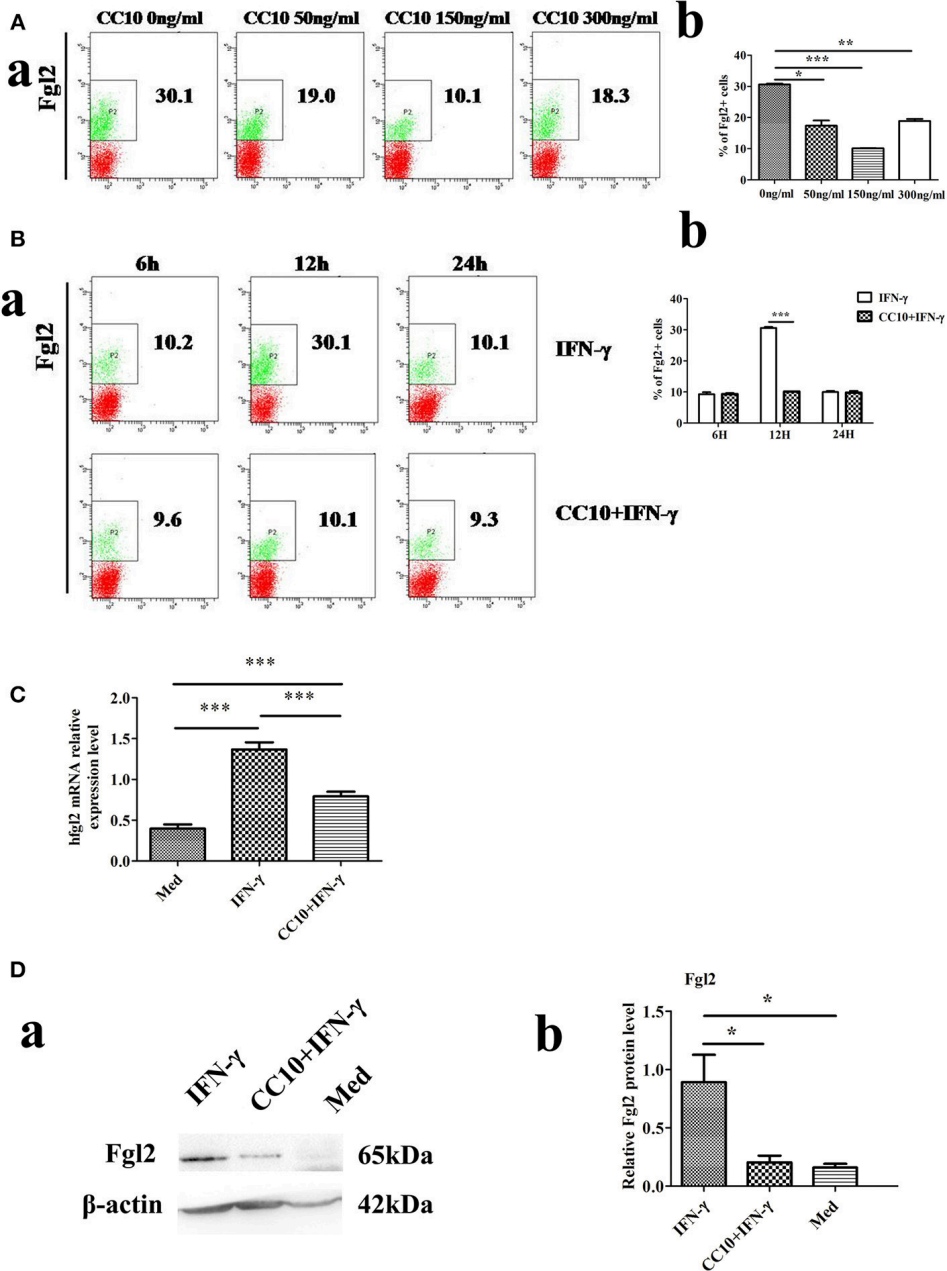
CC10 reduces expression of Fgl2 in THP-1 cells. THP-1 cells (2 × 10^6^) were treated with 100 ng/ml phorbol 12-myristate 13-acetate (PMA) for 48 h to induce differentiation toward adherent macrophage-like cells. The CC10 group was supplemented with CC10 protein (150 ng/mL). After 2 h of stimulation, IFN-γ (10 ng/ml) was added to the cells. The cells were cultured for 12 h before they were collected for flow cytometric analyses, western blot and qPCR studies. **(A)** Flow cytometry analysis of Fgl2 expression on THP-1 cells treated with CC10 protein at different concentrations. We examined the effect of increasing doses of CC10 protein (0, 50, 150, and 300 ng/mL) on IFN-γ induced Fgl2 expression in THP-1 cells for 12 h. 150 ng/mL CC10 protein had the strongest inhibitory effect on Fgl2 expression among these doses. **(a)** Representative flow cytometric dot plots are shown. **(b)** Percentage of positive cells that expressed Fgl2 in THP-1 cell populations. Data shown are means and standard error (*n* = 6/group). All results were representative of three independent experiments. *P < 0.05, **P < 0.01, and ***P < 0.001 for the CC10 protein group compared with the untreated group. **(B)** Flow cytometry analyses of Fgl2 expression in THP-1 cells treated with CC10 protein at different times. We examined the effects of increasing exposure to CC10 protein (6, 12, and 24 h) on IFN-γ induced Fgl2 expression in THP-1 cells at a dose of 150 ng/mL. CC10 protein incubated for 12 h had the strongest inhibitory effect on Fgl2 expression among these periods. **(a)** Representative flow cytometric dot plots are shown. **(b)** Different durations of CC10 protein exposure are shown. Data shown are means and standard error (*n* = 6/group). Each experiment was repeated in triplicate. ***P < 0.001 for the CC10+IFN-γ group compared with the IFN-γ group. **(C)** CC10 reduced the expression of Fgl2 in THP-1 cells. Gene expression of *hFgl2* assessed by qPCR. Values are shown as means and standard error (*n* = 6/group). All results were representative of three independent experiments. ***P < 0.001. **(D)** CC10 reduced the expression of Fgl2 in THP-1 cells. Fgl2 expression levels were measured by western blotting. **(a)** Representative western blotting figures are shown. **(b)** Quantitative measurement of Fgl2 expression. Values are shown as means and standard error (*n* = 6/group). All results were representative of three independent experiments. *P < 0.05.

We examined the effect of increasing doses of CC10 protein (0, 50, 150, and 300 ng/mL) on IFN-γ-induced Fgl2 expression in THP-1 cells. CC10 treatment showed a 10.1% decrease in THP-1 cells compared to that in control after stimulation with 10 ng/mL IFN-γ for 12 h. CC10 protein inhibited Fgl2 expression between doses of 0 ng/mL and 300 ng/mL (Figure 4A). In particular, 150 ng/mL CC10 protein had the strongest inhibitory effect on Fgl2 expression among the doses, and we chose this dose for the following experiments. We explored the effect of different time points of stimulation with a concentration of 150 ng/mL CC10 protein. After stimulation with CC10 protein for 6, 12, and 24 h compared to the PBS control, the strongest inhibitory effect on Fgl2 expression was noted at 12 h; hence, we chose this time point for the following studies (Figure 4B). An increasing number of studies suggest that macrophages are the primary source of Fgl2. In order to ascertain that CC10 has a direct effect on macrophages, we treated THP-1 cells with recombinant CC10 and assessed the expression of Fgl2. Unlike in controls, IFN-γ induced a significant increase in Fgl2 expression. This effect was attenuated when cells were treated with CC10 protein (Figures 4C,D), revealing that CC10 directly reduces the levels of Fgl2 in macrophages.

**Figure 5:**
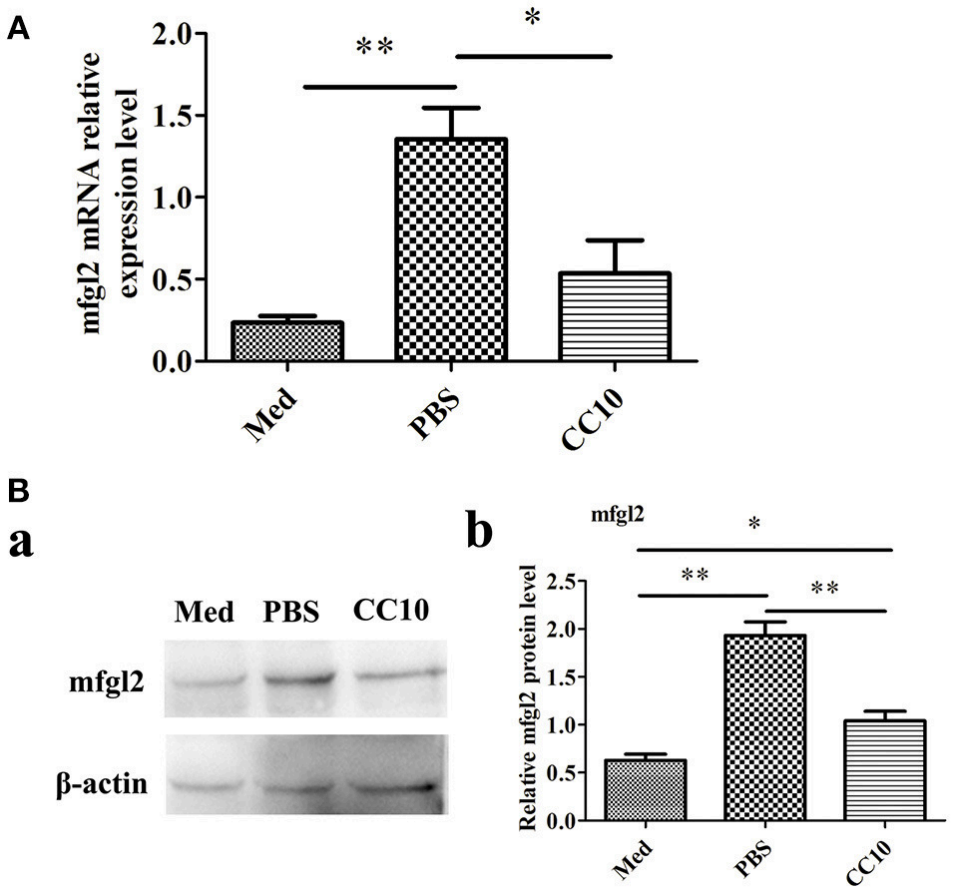
CC10 protein decreases MHV-3-induced Fgl2 expression in peritoneal exudates macrophages (PEMs). PEMs were exposed to 1,000 PFU MHV-3 for 4 h in the presence of recombinant murine CC10 protein or PBS. **(A)** PEMs were then collected for qPCR studies. Data are shown as means and standard error (*n* = 6/group). All results were representative of three independent experiments. ^*^P < 0.05 and ^**^P < 0.01. **(B)** PEMs were lysed and prepared for western blotting with a polyclonal anti-Fgl2 antibody. **(a)** Representative western blotting figures are shown. **(b)** Quantitative measurement of Fgl2 expression. Data are shown as means and standard error (*n* = 6/group). All results were representative of three independent experiments. ^*^P < 0.05 and ^**^P < 0.01.

To further explore the possibility that CC10 protein directly acts on macrophages, we infected murine PEMs with MHV-3 in the presence of recombinant CC10 and determined Fgl2 expression. Compared to levels in the controls, MHV-3-infected macrophages exhibited a significant increase in Fgl2 production, and this effect was abolished by using CC10 protein (Figures 5A,B), indicating that CC10 directly modulates Fgl2 production in macrophages.

### DNA Microarray Analysis Identified *HBP1* and *Fgl2* as Downregulated Genes After Stimulation With Cc10

**Figure 6:**
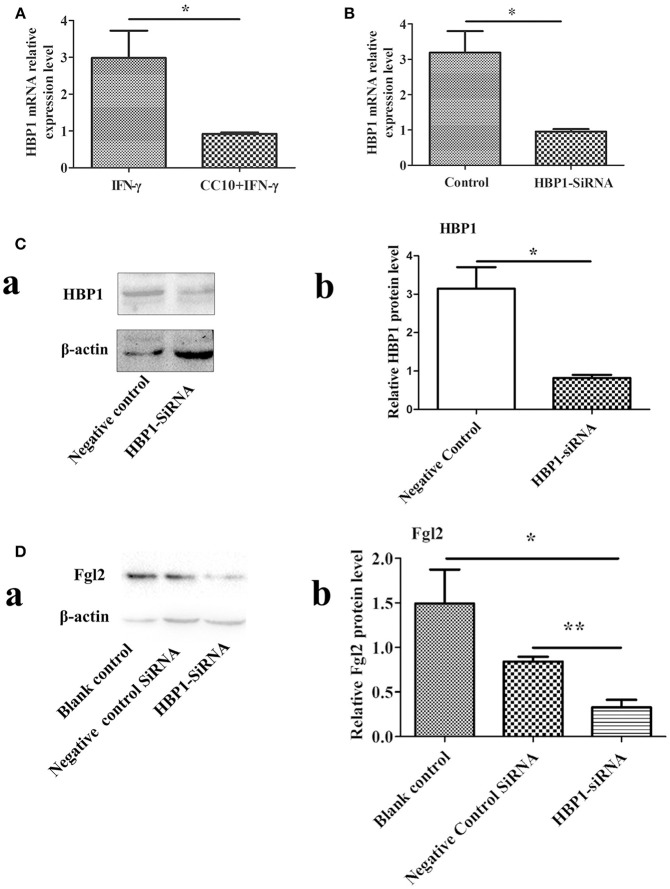
*HBP1* knockdown via siRNA resulted in decreased Fgl2 protein levels. **(A)** CC10 inhibited the expression of HBP1. To validate the results of DNA microarray experiments, genes were retested by means of qPCR. The expression of *HBP1* was downregulated after the addition of CC10 protein to the macrophages induced by PMA. Data are presented as means ± SD. *P < 0.05 for the CC10 group compared with the control group. **(B)** Effect of HBP1-siRNA on *HBP1* mRNA levels by qPCR. Transfection of HUVECs with HBP1-siRNA resulted in silencing of the *HBP1* gene. Data are presented as means ± SD. *P < 0.05 for the HBP1-siRNA group compared with the control siRNA group. **(C)** Effect of HBP1-siRNA on HBP1 protein by western blot. **(a)** Representative western blotting figures are shown. **(b)** Quantitative measurement of Fgl2 expression. Data are shown as means and standard error (*n* = 6/group). All results were representative of three independent experiments. *P < 0.05. **(D)** HBP1-siRNA resulted in decreased protein levels of Fgl2. Negative control is control siRNA. All results were representative of three independent experiments. **(a)** Representative western blotting figures are shown. **(b)** Quantitative measurement of Fgl2 expression. Data are shown as means and standard error (*n* = 6/group). All results were representative of three independent experiments. *P < 0.05 and **P < 0.01.

In order to determine genes that were downregulated after stimulation by CC10 protein, we used DNA microarray analysis to screen for differentially expressed genes. THP-1 cells were cultured and PMA was added to induce differentiation into macrophages. The production of Fgl2 was stimulated by IFN-γ. The experimental group was treated with CC10 protein for microarray detection of differentially expressed genes. The results showed that the most obviously downregulated genes were *UBE2W, HECTD1, MIR612, ATRX, SOX4, HBP1*, and *Fgl2* (Supplementary Table 1). And then these genes were tested by qPCR. However, *UBE2W, HECTD1, MIR612, ATRX*, and *SOX4* was not differentially expressed by qPCR, while HBP1 and fgl2 were still down-regulated genes. DNA microarray analysis identified *HBP1* as a down-regulated gene involved in the pathological processes of the regulation of CC10. Recently, very limited studies have explored the role of HBP1 in FH. Nevertheless, the mechanistic functions of HBP1 in FH remain largely unexplored. Therefore, we selected this gene for further study. qPCR analysis confirmed that mRNA levels of *HBP1* were significantly decreased in THP-1 cells after CC10 protein stimulation compared to that in the PBS control group (Figure 6A).

### HBP1 Promotes the Expression of Fgl2

We knocked down *HBP1* using HBP1-siRNA. Then, transfection of HBP1-SiRNA into HUVECs was detected by qPCR and western-blotting methods. As expected, *HBP1* knockdown led to significantly decreased expression of HBP1 (Figures 6B,C). Furthermore, *HBP1* knockdown impaired expression of *Fgl2* (Figure 6D), suggesting that HBP1 was able to activate Fgl2.

### CC10 Protein Inhibits Fgl2 Expression via HBP1

**Figure 7:**
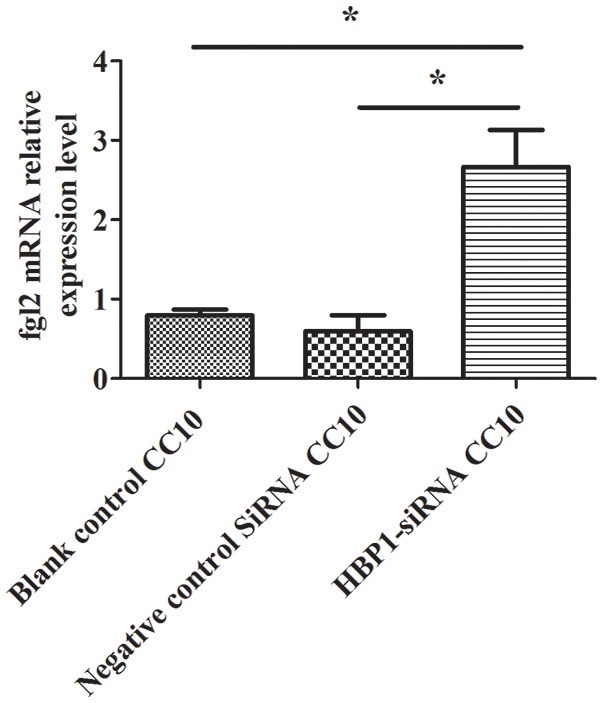
CC10 protein inhibits the expression of Fgl2 via HBP1. Human umbilical vein endothelial cells (HUVECs) were cultured. Transfection of HUVECs with HBP1-siRNA resulted in silencing of the *HBP1* gene. IFN-γ was added to the HUVECs after stimulation with CC10 protein (150 ng/ml) for 2 h. Compared to the expression in the blank control and control siRNA group, the expression of *Fgl2* in the HBP1-SiRNA group increased after stimulation with CC10 protein as determined by qPCR assays. It is suggested that CC10 may inhibit the expression of *Fgl2* via HBP1. Gene expression of *Fgl2* assessed by qPCR. Values are shown as means and standard error (*n* = 6/group). All results were representative of three independent experiments. *P < 0.05.

HBP1-SiRNA was used to transfect HUVECs. Then, IFN-γ was added to induce the expression of *Fgl2* followed by stimulation with CC10 protein (150 ng/ml) after 2 h. Finally, we explored the expression of *Fgl2* by qPCR. The results showed that HBP1-SiRNA treatment abrogated the inhibitory effect of CC10 on *Fgl2* expression in HUVECs (Figure 7). That is to say, CC10 could suppress *Fgl2* expression in macrophages. Such an effect may be mediated by the transcription factor HBP1.

## Discussion

It is well-known that CC10 protein can suppress the immune response. In animal models of allergic diseases of the respiratory tract, most of evidences confirm this inhibition (26). Its function in FH has not been investigated yet. Here, we used a murine FH model established by MHV-3 infection to explore the effects of CC10 in this disease process. To determine the role of CC10 in the pathogenesis of FH, CC10 protein was injected into a mouse FH model established by MHV-3 infection. MHV-3-induced liver injury in CC10-treated mice occurred rarely and the areas of lesions were much fewer than those in saline-treated control mice. In summary, these results suggested that CC10 could reduce pathological liver damage in this FH model together with lower mortality rates followed by MHV-3 infection.

MHV-3 induced fulminant viral hepatitis progresses rapidly and infected mice die within 3–5 days. Previous studies suggested fgl2 played a vital role in this process with a 15–40% increase of survival when fgl2 was deleted (12, 15, 27, 28). Multiple inflammatory factors or mediators including TNF-α and IFN-γ, IL-1β and C5aR have been demonstrated to promote FH progression with significant discrepancies between liver damage and survival rate (29–32), which is accordant with our observation that CC10 substantially alleviated liver injury though survival rate improved mildly. The survival rate based on hours may be more accurate to examine the effect of CC10 on FH.

It is speculated that fgl2 can mediate lethality in MHV-3-induced FH. This is due to the fact that fgl2 induces the deposition of fibrinogen, which leads to activation of the coagulation cascade and induction of procoagulant activity (15). To determine whether the tissue necrosis was mediated by Fgl2 in CC10-treated mice following infection, Fgl2 expression was observed. Results suggested that the expression of Fgl2 was significantly increased in MHV-3-induced FH mice and CC10 treatment significantly reduced the production of Fgl2 in the infected liver and serum. In addition, decreased fibrinogen deposition was also observed in the livers of CC10-treated mice. Therefore, our research results strongly clarify that the lower mortality of CC10-treated mice after MHV-3 infection is due to the lower levels of Fgl2 and decreased fibrinogen deposition.

Indeed, it has been reported that Fgl2 is expressed on macrophages, and the expression of Fgl2 is believed to be induced by IFN-γ and TNF-α (22). Cultured THP-1 cells activated by IFN-γ or IL-2 have been demonstrated, with induction of Fgl2 expression and enhanced activation of human prothrombin (23). Therefore, in this study, we explored this cell line to investigate the modulation of CC10 on Fgl2. Surprisingly, we found that CC10 directly inhibited IFN-γ-induced Fgl2 expression in THP-1 cells. As we know, IFN-γ has proved to be the main cytokine that leads to the development and progression of FH. Also, it was shown that IFN-γ might exert its own proinflammatory biological function through enhancing Fgl2 expression. Therefore, in our study, CC10 might counter the effect of IFN-γ in the setting of FH, which substantiates its role in FH. These results demonstrated that CC10 regulates the expression of Fgl2 in macrophages.

In the current study, we used co-immunoprecipitation to analyze binding between CC10 and Fgl2. In this study, we investigated possible protein-protein interactions between CC10 and Fgl2 *in vitro*. The Chinese hamster ovary (CHO) cells transfected with pcDNA3.1-hCC10 and pcDNA3.1-hFgl2. Cellular proteins were immunoprecipitated with anti-CC10 antibody or anti-Fgl2 antibody. Immunoblotting was performed with anti-Fgl2 and anti-CC10 antibodies. Immunoprecipitation of protein extracts from pcDNA 3.1-CC10 and pcDNA3.1-Fgl2 co-transfected CHO cells with anti-Fgl2 or anti-CC10 antibody followed by western blotting with Fgl2 and CC10 antibodies indicated that CC10 did not co-immunoprecipitate with Fgl2, showing that there is no direct relationship between CC10 and Fgl2 (data not shown). The results showed that CC10 has no direct interaction with Fgl2.

From our previous study the gene of fgl2 contributed profoundly in MHV-3 induced fulminant hepatitis and is extensively expressed in macrophages and endothelium (12, 33). Our microarray indicated a CC10 down-regulated fgl2 expression and this is further confirmed by qPCR and Western blotting *in vivo* (peritoneal macrophages) and *in vitro* (THP-1, macrophage cell line). Therefore, it is reasonable to focus on macrophages to display the effect of CC10 on fgl2 expression and eventually mice survival. We entirely agree there may be other possibilities for a protective effect of CC10 to contribute to the disease process. This is worth further studies. The potential receptor of CC10 has not been revealed yet. Our previous study have demonstrated that CC10 have effect of dendritic cells in allergic rhinitis (34). In this research, we evaluated the effect of CC10 on macrophages functions and found Fgl2 was substantially down-regulated upon CC10 treatment, therefore, we speculate that potential CC10 receptor may be also expressed on macrophages. The potential target of CC10 on other immune cells cannot be excluded.

DNA microarray analysis is one of the most powerful approaches for the potential identification of unexpected genes involved in pathogenic processes. By using this approach, HMG-box transcription factor 1 (*HBP1*) was found to be one of the most downregulated genes after CC10 treatment of THP-1 cells. HBP1 is a well-described transcriptional repressor that modulates expression of genes involved in cell cycle progression. In a recent study, it was found that *HBP1* is a direct target of miR-21 and confirmed that HBP1 modulates the inhibitory function of miR-21-ASO in hepatosteatosis and carcinogenesis simultaneously (23). HBP1 is an endogenous inhibitor of the Wnt signaling pathway in both normal and cancer cells. The tumor suppressor role of HBP1 has been reported in some malignancies, such as oral cancer and glioma (35). However, an association between HBP1 and Fgl2 has not been investigated yet. The current study clearly demonstrated that CC10 protects against MHV-3 induced FH via suppression of Fgl2 expression. Such effects might be mediated by HBP1. However, the functional status of HBP1 in the CC10 pathway requires further research, and such studies are conducting in our laboratory.

In conclusion, we demonstrated that CC10 could limit the immunopathological damage in MHV-3-induced FH mice. Our results suggest that enhancing CC10 expression by an immunotherapeutic approach might be an effective treatment for FH.

## Author Contributions

HY performed all the described experiments and wrote the manuscript. YL assisted with some experiments, analyzed experimental results, and edited the manuscript. HW analyzed experimental results. XW reviewed and edited the manuscript. JH, WY, DX, XL, GS, and QN provided experimental help and design.

### Conflict of Interest Statement

The authors declare that the research was conducted in the absence of any commercial or financial relationships that could be construed as a potential conflict of interest.

## Supplementary Material

Supplementary Figure S1 Fgl2 deficiency attenuates MHV-3-induced hepatitis. (JPG). **(A)** Histopathology of liver tissues (H&E staining; original magnification, × 200, *n* = 6/group) at 72 h post-MHV-3 infection was evaluated in MHV-3–infected wild-type(WT) BALB/cJ mice and Fgl2 knockout(Fgl2 KO) mice. Livers were collected from WT mice **(a)** and Fgl2 KO mice **(b)** at 72 h after MHV-3 infection. Arrows point to necrotic regions with inflammation. **(B)** Serum ALT levels from WT mice and Fgl2 KO mice at 72 h after MHV-3 infection (*n* = 6/group). **(C)** Serum AST levels from WT mice and Fgl2 KO mice at 72 h after MHV-3 infection (*n* = 6/group). Values represent means and standard error of three independent experiments performed in triplicate. ^**^*P* < 0.01.

Supplementary Table 1 List of down-regulated genes by DNA microarray. In order to determine genes that were downregulated after stimulation by CC10 protein, we used DNA microarray analysis to screen for differentially expressed genes. Briefly microarray analysis was used to screen changes in genome-wide gene expression patterns in THP-1 cells with or without CC10 protein. The changes in over 47,000 human gene expression patterns were assessed using Affymetrix gene microarrays (Human Genome U133 Plus 2.0) (CapitalBio Co. Ltd., Beijing, China). Three replicates were used for microarrays analysis. THP-1 cells were cultured and PMA was added to induce differentiation into macrophages. The production of Fgl2 was stimulated by IFN-γ. The experimental group was treated with CC10 protein for microarray detection of differentially expressed genes. The results showed that the most obviously downregulated genes were *UBE2W, HECTD1, MIR612, ATRX, SOX4, HBP1*, and *Fgl2*.

## Abbreviations

FH: fulminant hepatitis
MHV-3: murine hepatitis virus strain 3
Fgl2: Fibrinogen-like protein 2
CC10: Clara cell 10 KDa protein
ALF: acute liver failure
PFU: plaque-forming units
PBS: phosphate-buffered saline
ALT: alanine aminotransferase
AST: aspartate aminotransferase
PCA: pro-coagulant activity
HRP: horseradish peroxidase
TUNEL: terminal deoxynucleotidyl transferase dUTP nick end labeling.
