# Supplementary figures and images for: Clara Cell 10 kDa Protein Alleviates Murine Hepatitis Virus Strain 3-Induced Fulminant Hepatitis by Inhibiting Fibrinogen-Like Protein 2 Expression
Source: Front Immunol. 2018 Dec 13;9:2935. doi: 10.3389/fimmu.2018.02935 (PMC6300492; doi:10.3389/fimmu.2018.02935)

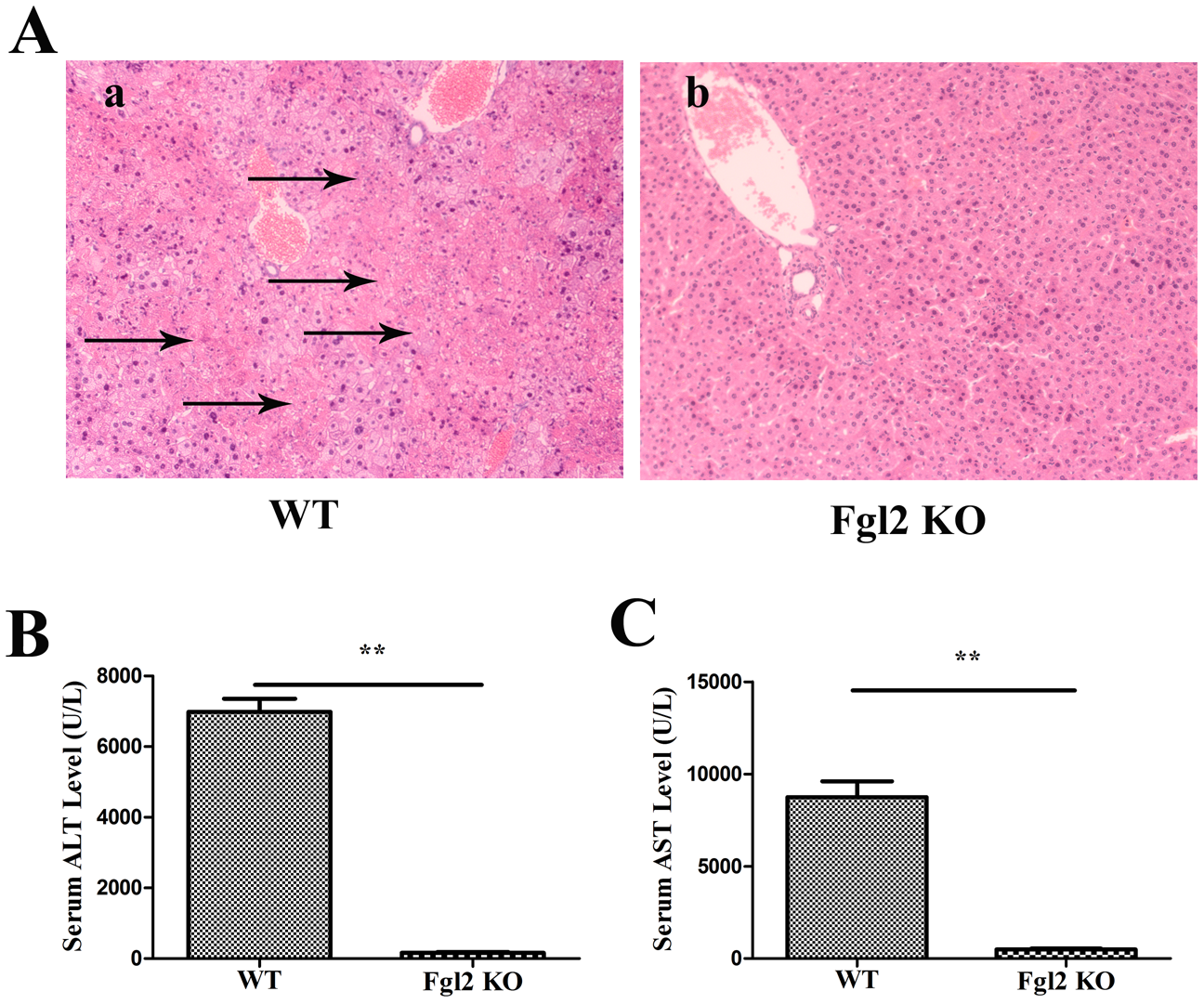

Supplement: Supplementary Figure S1 — Fgl2 deficiency attenuates MHV-3-induced hepatitis. (JPG). (A) Histopathology of liver tissues (H&E staining; original magnification, × 200, n = 6/group) at 72 h post-MHV-3 infection was evaluated in MHV-3–infected wild-type(WT) BALB/cJ mice and Fgl2 knockout(Fgl2 KO) mice. Livers were collected from WT mice (a) and Fgl2 KO mice (b) at 72 h after MHV-3 infection. Arrows point to necrotic regions with inflammation. (B) Serum ALT levels from WT mice and Fgl2 KO mice at 72 h after MHV-3 infection (n = 6/group). (C) Serum AST levels from WT mice and Fgl2 KO mice at 72 h after MHV-3 infection (n = 6/group). Values represent means and standard error of three independent experiments performed in triplicate. **P < 0.01. [file Image_1.TIF]
