# Supplementary material for: Clara Cell 10 kDa Protein Alleviates Murine Hepatitis Virus Strain 3-Induced Fulminant Hepatitis by Inhibiting Fibrinogen-Like Protein 2 Expression
Source: Front Immunol. 2018 Dec 13;9:2935. doi: 10.3389/fimmu.2018.02935 (PMC6300492; doi:10.3389/fimmu.2018.02935)
Supplement: Supplementary Table 1 — List of down-regulated genes by DNA microarray. In order to determine genes that were downregulated after stimulation by CC10 protein, we used DNA microarray analysis to screen for differentially expressed genes. Briefly microarray analysis was used to screen changes in genome-wide gene expression patterns in THP-1 cells with or without CC10 protein. The changes in over 47,000 human gene expression patterns were assessed using Affymetrix gene microarrays (Human Genome U133 Plus 2.0) (CapitalBio Co. Ltd., Beijing, China). Three replicates were used for microarrays analysis. THP-1 cells were cultured and PMA was added to induce differentiation into macrophages. The production of Fgl2 was stimulated by IFN-γ. The experimental group was treated with CC10 protein for microarray detection of differentially expressed genes. The results showed that the most obviously downregulated genes were UBE2W, HECTD1, MIR612, ATRX, SOX4, HBP1, and Fgl2. [file Table_1.DOC]

**Table 1：List of down-regulated genes by DNA microarray.**

| **Gene Symbol** | **Gene Title** |
| --- | --- |
| **HBP1** | **HMG-box transcription factor 1** |
| **FGL2** | **fibrinogen-like 2** |
| UBE2W | ubiquitin-conjugating enzyme E2W (putative) |
| HECTD1 | HECT domain containing E3 ubiquitin protein ligase 1 |
| ATRX | alpha thalassemia/mental retardation syndrome X-linked |
| MIR612 | microRNA612 |
| SOX4 | SRY (sex determining region Y)-box 4 |
